# Exploring the therapeutic potential of tDCS, TMS and DBS in overcoming tobacco use disorder: an umbrella review

**DOI:** 10.3934/Neuroscience.2024027

**Published:** 2024-10-23

**Authors:** Graziella Orrù, Marina Baroni, Ciro Conversano, Angelo Gemignani

**Affiliations:** Department of Surgical, Medical, Molecular & Critical Area Pathology, University of Pisa, via Savi, 10, 56126, Pisa, Italy

**Keywords:** umbrella review, non-invasive brain stimulation, tDCS, rTMS, TMS, DBS, nicotine dependence, tobacco use disorder

## Abstract

The purpose of the present study was to investigate the effects of neuromodulation techniques, including transcranial direct current stimulation, transcranial magnetic stimulation, and deep brain stimulation, on the treatments of nicotine dependence. Specifically, our objective was to assess the existing evidence by conducting an umbrella review of systematic reviews. The quality of the included studies was evaluated using the standardized tools designed to evaluate systematic reviews. The PubMed/MEDLINE database was queried for systematic reviews, and yielded 7 systematic reviews with a substantial sample size (N = 4,252), some of which included meta-analyses. A significant finding across these studies was the effectiveness of neuromodulation techniques to reduce nicotine cravings and consumption, through the evidence remains not yet conclusive. A significant efficacy of transcranial direct current stimulation and repetitive transcranial magnetic stimulation that targeted the dorsolateral prefrontal cortex was found, as well as the lateral prefrontal cortex and insula bilaterally, on smoking frequency and craving. Moreover, smoking behaviors may also be positively affected by the use of deep brain stimulation (DBS) targeting the nucleus accumbens. In conclusion, neuromodulation approaches hold promise as effective treatments for tobacco use disorder. Nonetheless, further research is required to comprehensively understand their effectiveness and to determine if combining them with other treatments can aid individuals to successfully quit smoking.

## 1. Introduction

Tobacco use disorder is a chronic relapsing condition characterized by cravings and uncontrollable use (compulsive use). Approximately 47.1 million individuals in the United States, which accounts for 19.0% of the population, currently engage in tobacco products [1]. In general terms, tobacco consumption is associated with 5 million deaths annually worldwide [2], including coronary heart disease, and cancers of the lung and upper airways as the main causes of death, amongst others [3] (for more details refer to table 1).

In this context, smoking cessation mitigates the likelihood of developing chronic diseases, thus enhancing life expectancy. Unfortunately, quitting smoking and maintaining abstinence pose substantial challenges due to the complex nature of tobacco dependency, thereby encompassing behavioral, cognitive, and physiological factors. In fact, despite numerous attempts, a significant percentage of tobacco smokers fail to achieve long-term cessation [4], with high relapse rates [5].

Different smoking cessation aids [2] (refer to table 3 for an exhaustive list of pharmacotherapies) are commonly used, including the following: i) first-line therapies (i.e., nicotine replacement therapy, nicotine patch); ii) non-nicotine products (i.e., bupropion, varenicline); iii) second-line therapies (i.e., clonidine, nortriptyline); and iv) non-pharmacological treatments (i.e., counselling, educational programmes, cognitive therapy). Moreover, although there is growing evidence of success rates, the definitive cessation rate is still relatively low, at approximately 15–25% [2],[6], thus leading to high relapse rates [7].

In recent years, despite the many factors that can influence the effectiveness of the aforementioned techniques (e.g., genetics factors, neurobiological profiles and outcomes expectations) [8]–[11], the efficacy of neuromodulation techniques in reducing the smoking frequency among patients with nicotine dependence has garnered significant attention. In fact, in the field of addiction research, several empirical research studies have pointed out the effectiveness of non-invasive brain stimulation (NIBS) in the context of substance use disorder [12]–[14]. In particular, the effects in reducing the frequency of use and craving for several substances, both legal (e.g., nicotine and alcohol) and illegal (e.g., cannabis and opioids), were observed [12]–[14]. This interest is supported by the broader application and success of these methodologies in various psychiatric and neurological populations. For instance, *repetitive transcranial magnetic stimulation* (rTMS), *transcranial direct current stimulation* (tDCS) and *deep brain stimulation* (DBS) have shown promising results in treating depression (i.e., tDCS [15], rTMS [16], DBS [17]), stroke rehabilitation (tDCS [18], rTMS [19],[20], DBS [21],[22]), movement disorders such as Parkinson's disease (tDCS [23], rTMS [24], DBS [25],[26]), cerebellar ataxia (tDCS [27], rTMS [28], DBS [29]), phantom limb (tDCS [30], rTMS [31], DBS [32]), and food craving (tDCS [33], rTMS [34], DBS [35]) by modulating neural activity and improving clinical symptoms and functional recovery. From a practical point of view, TMS and tDCS stimulate cortical regions (e.g., dorsolateral prefrontal cortex) by inducing an electrical current through a magnetic pulse and a low current intensity, respectively [36]. Furthermore, regarding the TMS, the use of specific coils (H coils) allows for not only superficial cortical areas to be stimulated, but also deeper brain areas [26]–[37]. This is particularly useful when there is a need for a wider stimulation of brain areas [36],[37]. Moreover, literature findings also pointed out that 10Hz-TMS enabled the functions of activities in terms of the basal ganglia in the reward process and the anterior midcingulate cortex among people with substance use disorder [38]. Regarding the tDCS, new types of montages, such as high definition-tDCS, have recently been introduced in order to make electric fields more focused [39]. Additionally, tDCS appeared to be able of stimulating the fronto-basal ganglia inhibitory network [38], which plays a crucial role in several disorders, including problematic substance use [40], thus not limiting its effects solely to cortical areas. On the other hand, DBS stimulates deeper brain areas such as the nucleus accumbes by implanting bipolar electrodes directly into the targeted areas [36].

Although the different neuromodulation modalities demonstrate their positive effect, the comparative efficacy of these methods remains unclear. In this context, a collection of systematic reviews and a network meta-analysis of randomized (NMA) controlled trials (RCTs) can provide valuable insights into the comparative benefits and safety of the different interventions.

This study aims to investigate the efficacy and safety of different neuromodulation methods in individuals with nicotine dependence, thereby focusing on changes in the smoking frequency and acceptability (dropout rates) as the primary outcomes.

### 1.1. A brief overview of nicotine's impact on brain circuits and neurotransmitter release

Tobacco use disorder is predominantly the result of the pharmacological effects of nicotine, despite most of the harmful toxicity of smoking being attributed to other components. Nicotine represents the main addictive agent in tobacco smoke, thereby exerting its effects primarily mediated by the brain through a complex interplay of neurobiological mechanisms. Similar to other highly addictive substances, nicotine stimulates the reward circuits that develop to enhance the desire for natural rewards. It has the ability to enter the blood vessels in the brain and attach to specific receptors, namely the neuronal nicotinic acetylcholine receptors (nAChRs); this attachment influences the release of many neurotransmitters in the brain, such as acetylcholine, serotonin, dopamine, glutamate, and γ-aminobutyric acid (GABA) (i.e., [41]). In this context, numerous studies (i.e., [42]–[45]) have suggested that dopamine appeared to play a crucial role since nicotine increased the firing of dopamine neurons, thus promoting the release of dopamine into the nucleus accumbens (NAcc) in the ventral striatum. In general terms, the ventral striatum is well established to contribute to motivated behavior [46] and is widely recognized as a key-region associated with rewards. It has been directly linked to the processing and learning of rewards in both animal and human studies, rendering it a crucial area involved in substance use disorder. Furthermore, the release of other neurotransmitters, such as norepinephrine and endorphins, amongst others, contributes to the diverse behaviors linked with nicotine use [43].

In terms of nicotine-related brain activity, Stein and colleagues (1998) [47] showed that nicotine caused an increase in the blood oxygenation level dependent (BOLD) functional magnetic resonance imaging (fMRI) signal in various regions of the brain, such as the insula, cingulate cortex, dorsolateral, orbital, and medial prefrontal cortices, as well as parts of the temporal and occipital cortices. Furthermore, the NAcc, amygdala, hypothalamus, and thalamic nuclei were also affected. These results are supported by additional investigations (i.e., [48]). Overall, these outcomes align with the activation of corticobasal ganglia-thalamic brain circuits that are associated with substance use disorder.

Other empirical research has investigated the impact of long-term cigarette smoking on structural brain measurements; in this regard, using high-resolution structural MRI, Brody et al. (2004) [49] highlighted that smokers have reduced grey matter (GM) volumes and densities in the bilateral prefrontal cortex (PFC) and left dorsal anterior cingulate (dACC) compared to non-smokers. Analogue findings have been drawn by Gallinat et al. (2006) [50], who found that smokers presented lower GM (volume and density) compared to non-smokers in the following brain areas: anterior cingulate cortex (ACC), PFC, orbitofrontal cortex, occipital and temporal lobes, thalamus, and cerebellum. A recent meta-analysis conducted by Yang et al. (2020) [51] revealed that chronic smokers exhibited significant losses in their GM volume in both the PFC and left insular, and experienced an increase in GM in the right lingual cortex and left occipital cortex. While some of the reported findings are corroborated by different research, other findings are contradictory. For example, in this regard, Zhang et al. (2011) [52] found that smokers exhibited a greater density of GM in the left insular cortex. This finding supports the idea that the insula may be implicated in nicotine use disorder (i.e., [53],[54]). Conclusive evidence about the insula is not yet possible, as different studies have found conflicting results, therefore prompting the need for further investigations.

## 2. Material and methods

### 2.1. Literature search, inclusion and exclusion criteria

To determine suitable publications for inclusion, a search was performed on the PubMed database on July 18^th^, 2024, using the specified search terms: “*transcranial direct current stimulation*” OR “*tdcs*” AND “*nicotine*” OR “*tobacco*”; “repetitive *transcranial magnetic stimulation*” OR “*rTMS*” AND “*nicotine*” OR “*tobacco*”; “*deep brain stimulation*” OR “*DBS*” AND “*nicotine*” OR “*tobacco*”. In addition to the search criteria described above, we also hand cross-referenced the publication list referenced by the studies we retained to guarantee that no relevant articles were excluded. Additionally, no language or date restrictions were applied. The only filter that was applied was the article type relevant to a specific criterion for umbrella review, which was restricted solely to systematic reviews/meta-analysis.

During the selection phase, systematic reviews were included if the following inclusion criteria were met: (a) systematic reviews, concluding or not with meta-analysis reporting results on the application of TMS, DBS, and/or tDCS in the context of tobacco consumption; and (b) results exclusively derived from systematic reviews related to tobacco use. We did not include all systematic reviews and/or meta-analysis conducted on non-human individuals, as well as duplicates, irrelevant studies, and those that did not contain at least one unique article (i.e., not duplicated in the other works).

Additionally, the findings obtained from systematic reviews that examined neurologic and psychiatric disorders, cognitive domains, or other types of substance use disorder such as cannabis, cocaine, methamphetamine, opioids, and alcohol were excluded to avoid any confounds. After reading the title and abstract, the irrelevant studies were excluded. The authors (G.O. and M.B.), who were blinded to each other's findings, screened the titles and abstracts. Subsequently, full-text articles were screened. The same authors independently extracted data from the included studies and disagreements were resolved through discussion until a consensus was reached. To evaluate the methodological quality of the reviewed studies, we employed the “*Revised Assessment of Multiple Systematic Reviews*” (R-AMSTAR [55]) and the “*A MeaSurement Tool to Assess Systematic Reviews 2*” (AMSTAR-2 [56],[57]) tools to determine the specific scores and to perform a quality evaluation, respectively.

### 2.2. Methodological quality

The overall methodological quality of the eleven included systematic reviews and meta-analysis that were assessed through both the AMSTAR-2 and the R-AMSTAR tools is summarized in **Table 1**. We employed both tools to ensure a thorough and robust evaluation of the systematic reviews included in our analysis. R-AMSTAR provides a detailed assessment with refined criteria of the AMSTAR [55],[58], while AMSTAR-2 enhances the evaluation by incorporating additional items and modifications suitable for both randomized and non-randomized studies [56],[57]. Using both tools allows us to comprehensively appraise the methodological quality and reliability of the systematic reviews, thus ensuring the highest standard of rigor in our research.

The AMSTAR-2 is composed of 16 items and assesses seven different critical domains. The score may be categorized as either “*High*” (i.e., no or one non-critical weakness), “*Moderate*” (i.e., more than one non-critical weakness), “*Low*” (i.e., one critical flaw with or without non-critical weaknesses), or “*Critically low*” (i.e., more than one critical flaw with or without non-critical weaknesses). On the other hand, the R-AMSTAR is characterized by the eleven original items of AMSTAR [58] that can be each scored from 1 to 4 points, thus reaching a minimum score of 11 points and a maximum score of 44 points (“*Low*”: 11–22 points; “*Medium*”: 23–33 points; “*High*”: 34–44 points). The instrument was created in order to add a quantifiable assessment of the quality of systematic reviews [55],[59]. Two different research groups created the AMSTAR-2 and R-AMSTAR; however, most of the original studies used the former (the most recent) compared to the latter [55]–[57],[59].

## 3. Results

**Figure 1.**
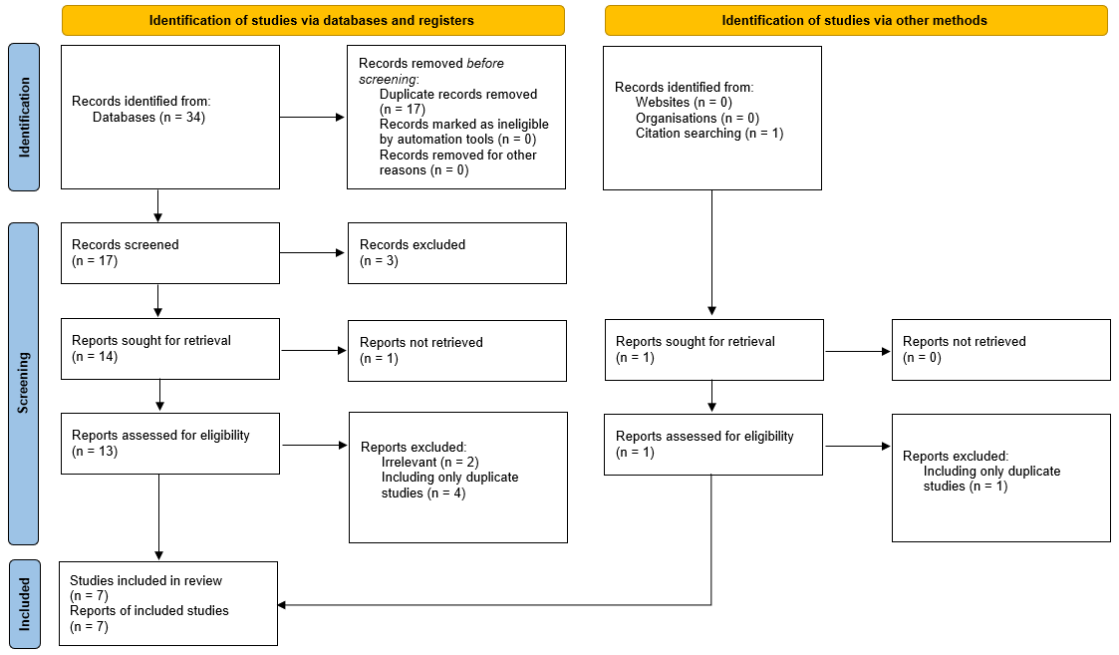
Preferred Reporting Items for Systematic Reviews and Meta-analyses (PRISMA) flow diagram showing the literature search, screening process, study selection and results.

We obtained a total of 34 hits, of which we retained 7 (TMS: 4; tDCS: 3) studies according to our inclusion and/or exclusion criteria. The studies selected (**Figure 1**) satisfied the preferred reporting items for systematic Review (PRISMA) [60].

A synopsis of the studies aims, results, and the intervention applied (TMS, tDCS and/or DBS) are shown in **Table 1**.

**Table 1.** Description of the studies and the AMSTAR-2 and R-AMSTAR quality/scores.

**Study**	**Intervention**	**Number of studies included/RCT**	**Total sample size (active/sham)**	**Mean age (years)/women (%)**	**Treatment duration**	**AMSTAR-2 Quality**
Kang, Kim, Kim, 2019 [61]	tDCS	7 RCT and 5 Crossover	N=392	33.83; 34.74%	range = 1-10 sessions	Critically low
						
						**R-AMSTAR Quality/Scores**
						High/34

*Aim of the study*: effects of tDCS on symptoms of nicotine dependence in treatment-seeking smokers						
*Main results*: random-effects model meta-analyses showed that tDCS had substantial favourable effects on seven cue-provoked craving comparisons (effect size=0.422; P=.004) and eight smoking intake comparisons (effect size=0.557; P=.004). The results of the moderator variable analysis showed that the application of anodal-tDCS on the right DLPFC had a significant favourable impact on cue-provoked craving with low heterogeneity across studies.						

Mehta et al., 2024 [62]	rTMS/tDCS/DBS	94 (NR = 28) (rTMS = 51; NR = 16 tDCS = 36; NR = 11 DBS = 7; NR = 1)	4,036 (NR = 1,239) (rTMS = 2,406; NR = 781 tDCS = 1,589; NR = 448 DBS = 48; NR = 10)	NA	range= 2-20 sessions	Critically low
						
						**R-AMSTAR Quality/Scores**
						High/35

*Aim of the study*: efficacy of neuromodulation in improving behavioural outcomes in substance use disorders						
*Main results*: active rTMS reduces tobacco craving and/or cigarette consumption compared to sham rTMS, with the exception of a few studies that did not find significant effects. The most effective rTMS protocols involve multiple sessions targeting the left dorsolateral prefrontal cortex (DLPFC) or bilateral DLPFC, with frequencies of 10 Hz or 20 Hz.						
Chan et al., 2023 [63]	tDCS	43	N=611	31.36; 35.67%	range= 1-10 sessions	Low
						
						**R-AMSTAR Quality/Scores**
						High/36

*Aim of the study*: efficacy of tDCS in reducing craving for different substances (alcohol, opioids, methamphetamine, cocaine, tobacco, and cannabis)						
*Main results*: tDCS has an effect in reducing craving levels among tDCS group compared to sham group (SMD = -1.07; p = .002)						

Petit et al., 2022 [64]	rTMS/tDCS	7 (rTMS = 6 tDCS = 1)	N=699 (rTMS = 559 tDCS = 140)	NA; 29.7% (rTMS = NA; 43.7% tDCS = NA; 0%)	range= 4-20 sessions	Low
						
						**R-AMSTAR Quality/Scores**
						High/40

*Aim of the study*: efficacy of NIBS (rTMS and tDCS) on long-term smoking cessation						
*Main results*: compared to sham stimulation, NIBS (rTMS and tDCS) have a significant overall effect on sustained abstinence for 3–6 months after smoking cessation (RR= 2.39; 95% CI = 1.26–4.55; I^2^ = 40%; p = 0.008). By isolating the rTMS method, the effect on sustained abstinence was still significant compared to sham condition (RR=2.07; 95% CI = 1.08–3.98; I^2^ = 35%, p= 0.03). Subgroup analysis pointed out both a significant effect of excitatory rTMS targeting the left DLPFC (RR = 4.34; 95% CI = 1.69–11.18; I^2^ = 0%; p= 0.002) and of deep rTMS targeting the lateral prefrontal cortex and insula bilaterally (RR = 4.64; 95% CI = 1.61–13.39; I^2^ = 0%; p= 0.005).						

Hauer et al., 2019 [65]	rTMS	16 (HP=11; CP = 5)	N= 563 (HP=396; C = 167)	NA	range= 1-21 sessions	Critically low
						
						**R-AMSTAR Quality/Scores**
						Medium/29

*Aim of the study*: the use of rTMS on nicotine use and craving						
*Main results*: high frequency (HF) rTMS targeting the PFC may be considered a suitable tool for tobacco consumption and craving among the health population. Despite inconsistent literature results, HF-rTMS could be effective also among subjects with schizophrenia. Particularly, the use of deep rTMS and theta burst stimulation pointed out promising results on tobacco consume and craving.						
Zhang et al., 2019 [66]	rTMS	26 (NR = 9)	N= 748 (NR = 318)	NA	range= 1-16 sessions	Critically low
						
						**R-AMSTAR Quality/Scores**
						High/37

*Aim of the study: efficacy of rTMS in reducing consumption and craving for different substances (alcohol, nicotine, and illicit drugs)*						
*Main results*: compared to sham stimulation, excitatory rTMS targeting left DLPFC has a significant effect on craving (Hedges' g = -0.62; 95% CI = -0.89 to -0.35; P < 0.0001). Concerning the excitatory stimulation of the DLPFC, meta-regression highlighted a significant and positive association between the total number of stimulation pulses and effect size (p= 0.01).						

Shaheen et al., 2023 [67]	DBS	16 (NR = 2)	N=50 (NR = 11)	41.03; 20% (NR = 45.25; 0%)	NA	Critically low
						
						**R-AMSTAR Quality/Scores**
						High/34

*Aim of the study:* efficacy of DBS in the treatment of substance disorder (including nicotine use disorder) and reduction of relapse rates						
*Main results*: based on age and types of substance use disorder, subgroup and meta-regression analysis pointed out that DBS could be more effective for patients above 45 years of age, and for alcohol and opioid use disorder compared to nicotine use disorder.						

*Notes*: *CP*, clinical population; *DLPFC*, left dorsolateral prefrontal cortex; *DTMS*, deep transcranial magnetic stimulation; *FPT*, frontal-parietal-temporal area; *HF*, High frequency; *HP*, health population; *NAcc*, Nucleus Accubens; *Nacc-A*, Nucleus Accubens ablation*; NIBS*, Non-invasive brain stimulation; *NR*, Nicotine related; *SMD*, standardized mean difference; *PFC*, prefrontal cortex; *RR*, Risk Ratio.

### 3.1. Methodological quality: qualitative and quantitative assessment

The overall methodological quality of the twelve included systematic reviews and meta-analyses is summarized in **Table 1** (for further details, refer to the supplementary materials and consult **Table S1** and **S2**).

Based on the AMSTAR-2, eight studies were rated as “*Critically low*” and four as “*Low*”. However, due to the limited literature on the application of NIBS to smoking patterns, we decided to not exclude any of the retained studies. In most cases, the factors that contributed to a low confidence level were attributed to the lack of i) protocol registration (item 2), ii) a list of excluded studies (item 7), iii) and a report of funding sources for the included studies (item 10).

Conversely, considering the R-AMSTAR scoring, eight of the twelve included studies achieved a score that defined their methodological quality as “*High*” (34–44 points). The major identified weaknesses were related to the exclusion of papers based on their publication type (item 4) and the lack of both a list of excluded studies (item 5) and a publication bias analysis (item 10).

### 3.2. Transcranial Direct Current Stimulation

Three of the included systematic reviews and meta-analysis investigated the use of tDCS to manage several aspects of tobacco consumption (e.g., smoking intake/cessation and craving reduction). The systematic review by Petit and colleagues (2022) [64] was not included in this section considering that it only contained one study on tDCS which was already included in other works presented here. In most cases, the main stimulated area was the dorsolateral prefrontal cortex (DLPFC) (unilaterally or bilaterally); however, several studies also targeted different regions such as the frontal-parietal-temporal (FTP) area, left occipital lobe (OL), right supraorbital (R-SOB) area, and insula [61]–[63].

Regarding cigarette consumption and smoking behavior, several studies have highlighted the beneficial effects of tDCS on different aspects of tobacco use, including smoking cessation and craving reduction [61]–[63]. Notably, compared to the sham condition, anodal and/or cathodal tDCS (atDCS and ctDCS, respectively) applied over the DLPFC appeared to reduce cigarette consumption [61]. Notably, a significant effect was observed by applying atDCS over the right DLPFC [61]. Conversely, Mehta and colleagues (2024) [62] did not find any significant effects of active stimulation on craving and consumption compared to sham stimulation.

### 3.3. Transcranial Magnetic Stimulation

Of the seven systematic reviews included in our umbrella review, four studies examined the effect of TMS, such as *repetitive* TMS (rTMS), *deep* TMS (DTMS) and/or *high frequency* TMS (HF-TMS), on several aspects of smoking behaviors (e.g., consumption, craving, relapse, and abstinence) [62],[64]–[66]. The primary targeted area was the DLPFC. However, stimulation of other areas, such as the superior frontal gyrus (SFG), prefrontal cortex, and insula, was also observed [62],[64]–[66].

In regard to consumption behavior, studies have detected the efficacy of rTMS and DTMS in reducing the tobacco consumption frequency compared to the sham condition [65]–[67]. Additionally, several authors highlighted the effectiveness of rTMS in reducing cravings [62],[66] and promoting abstinence [64]. Regarding craving reduction, Mehta and colleagues (2024) [62] pointed out the greater efficacy of multiple stimulation sessions; for promoting abstinence, Petit et al., (2020) [64] observed the effect of non-invasive brain stimulations such as TMS and tDCS to sustain abstinence for 3-6 months following smoking cessation.

### 3.4. Deep Brain Stimulation (DBS)

Finally, although the current literature lacks a sufficient number of reviews to justify an umbrella review, we find it essential to discuss the effects of DBS on nicotine use disorder to provide a comprehensive overview of the application of neuromodulation techniques in this field. In line with this, Shaheen and colleagues' meta-analysis (2023) [67] pointed out that DBS targeting the NAcc had an effect on nicotine use disorder; however, a subgroup analysis showed no significant differences across different types of substance use disorder (alcohol, opioid, and nicotine), and highlighted a greater DBS effectiveness for alcohol and opioid use disorder compared to nicotine use disorder. Despite this, the study underscored the potential effects of DBS on smoking symptoms, thus laying the foundation for future targeted research.

## 4. Discussion

The results of this umbrella review highlight the potential of neuromodulation techniques, such as tDCS, TMS, and DBS, to treat nicotine use disorder. These interventions show promise in reducing cravings and cigarette consumption with specific protocols such 10-Hz rTMS over the left DLPFC and bifrontal tDCS.

Our study pointed out the efficacy of tDCS, rTMS, and DBS to treat several aspects of tobacco-related behaviors such as smoking frequency and intake, relapse, abstinence, and craving. Regarding the investigated neuromodulation approaches, most of the included studies focused on the application of tDCS and rTMS followed by DBS on different aspects of tobacco use (cigarette consumption and craving).

In terms of the tDCS efficacy, the included studies reported that a tDCS over the right DLPFC had a substantial effect on craving [61]. Moreover, the effectiveness of DLPFC-ctDCS on tobacco use and cue-provoked cravings was also observed [61]. Regarding the effectiveness of DLPFC-tDCS to reduce the smoking frequency, Tseng and colleagues (2022) have suggested that the improvement in DLPFC activity induced by the technique positively affects cognitive control [68],[69]. Cognitive control is a construct that plays a significant role in behavioral self-regulation, including smoking behaviors and habits [69]. In terms of cravings, given that cue-provoked cravings in smokers have been linked to impairments in executive functions, the effectiveness of DLPFC-tDCS may be attributed to the improvement in cognitive processes that this technique induces [61],[70]. Regarding rTMS, specific protocols such as HF-rTMS and 10-Hz rTMS over the left DLPFC have demonstrated a significant efficacy to reduce the smoking frequency and intake [65]. Furthermore, HF-rTMS was shown to be effective on cravings [65]. Petit et al. (2022) [64] highlighted the efficacy of DTMS, excitatory rTMS, and deep rTMS on cravings. Efficacy was observed in stimulating the DLPFC with atDCS, as well as the lateral prefrontal cortex and insula bilaterally targeted by deep rTMS [65]. As previously stated, rTMS stimulation over the DLPFC may be considerably effective in improving the executive functioning and cognitive control [61],[69],[70]. However, the effects of tDCS on cognitive control should be interpreted with caution while considering the conflicting data on this issue [70]. On the other hand, the efficacy on craving derived from the application of rTMS stimulation over the lateral PFC and a bilateral stimulation of the insula may be associated with their role in drug-seeking behaviors and the modulation of emotional and internal craving dimensions, respectively [72]–[74]. Finally, the effectiveness of neuromodulation on nicotine cravings and smoking cessation may be due to individual and neurobiological factors. Concerning the first ones, both outcomes' expectations and motivations to quit smoking may have a positive effect on neuromodulation effectiveness [9]–[10]. On the other hand, regarding neurobiological factors, significant outcomes may be related to the putative interactions between neural systems linked with problematic use of nicotine and the targeted areas stimulated by NIBS [74]. Specifically, the literature established that there is a dopaminergic hypofunction in the prefrontal cortex, which is an area deputed to counteract craving [75], among people with substance use disorder [76],[77], such that stimulating cortical excitability in the aforementioned brain region can result in a reduction of cravings by promoting smoking cessation.

Finally, the application of DBS for the treatment of tobacco use disorder has been explored in a limited number of studies [67], thus making difficult to draw definitive conclusions about its efficacy compared to other brain stimulation techniques such as rTMS and tDCS. Moreover, although significant differences in the effectiveness of this technique across different substance use disorders have emerged [67], it is possible to assume that DBS may be effective in this area. Specifically, considering the role of the NAcc in the reward system, the direct stimulation of this brain structure may be effective in both reducing cravings and contrasting relapses [78],[79].

The variability of the findings, particularly in the context of DBS investigations, suggests that individual differences and specific parameters of the stimulation protocols may play a crucial role in the effectiveness of the intervention.

In general terms, the heterogeneity of the results highlights the following: (i) the complexity of nicotine use disorder; (ii) the roles of specific individual factors including genetic predispositions, neurobiological profiles, environmental influences, as well as expectations and motivations; and (iii) an additional layer of complexity arises from the fact that the effects of NIBS are influenced by a variety of parameters, such as the type of stimulation, coil or electrode size, positioning, stimulation polarity, current density, intensity, spatial focality of the signal, and the number of sessions and their duration. All these variables may play a crucial role in determining the efficacy of neuromodulations techniques, thereby requiring more personalized treatment approaches. By giving an example, with the above factors, it might be useful to manage the administration of self-report questionnaires such as the “*Expectation Assessment Scale*” (EAS) [9],[80] or the “*Visual Analog Scale for the motivation to quit smoking*” [10],[81], as well as conduct a genetic pre-screening [11]. Finally, as highlighted in the work of Chan and colleagues (2023) [63], it could be useful to administer the “the Adverse Effects tDCS Questionnaire” at the end of the experiment or treatment to ameliorate scientific protocols and guidelines in the field by reducing the putative negative consequences linked with neuromodulation techniques.

## 5. Limitations and future perspectives

The present work suffered from a number of limitations. First, some of the systematic reviews and meta-analysis included in the umbrella review overlapped with the primary studies. This factor was taken into account during the interpretation and discussion to avoid overestimating the observed outcomes. Second, the low methodological quality of the included systematic reviews and meta-analyses (as assessed by AMSTAR-2 and R-AMSTAR) may have impacted methodological rigor. Therefore, the results should be interpreted with caution. Beyond that, future in-the-field studies will be necessary to obtain more consistent and robust findings. On the other hand, the present work represents an overview and a possible groundwork for further research on the use of NIBS to address problematic nicotine use by providing key data on commonly used methodologies and technique parameters.

## 6. Conclusions

The findings from this umbrella review indicated that tDCS, rTMS, and DBS appear to be promising tools to treat nicotine cravings and consumption. However, the results are not yet conclusive, and further research is needed to determine the optimal protocols and long-term efficacy of these techniques.

Future research should focus on optimizing stimulation parameters, exploring individual differences in response to treatment, and examining the potential synergistic effects of combining neuromodulation with other therapeutic approaches. Additionally, investigating the underlying neural mechanisms may offer insights into how these treatments can be tailored to maximize their effectiveness for individuals struggling with nicotine use disorder.

## Use of AI tools declaration

The authors declare they have not used Artificial Intelligence (AI) tools in the creation of this article.


