# Supplementary material for: Exploring the therapeutic potential of tDCS, TMS and DBS in overcoming tobacco use disorder: an umbrella review
Source: AIMS Neurosci. 2024 Oct 23;11(4):449–67. doi: 10.3934/Neuroscience.2024027 (PMC11712234; doi:10.3934/Neuroscience.2024027)
Supplement: Supplementary file 1 [file neurosci-11-04-027-s001.pdf]

AIMS Neuroscience

http://www.aimspress.com/journal/neuroscience

AIMS Neuroscience, 11 (4): 449–467.
DOI: 10.3934/Neuroscience.2024027
Received: 19 July 2024
Revised: 08 October 2024
Accepted: 18 October 2024
Published: 23 October 2024

Mini review

Supplementary Materials

Table S1. AMSTAR-2 scores of the systematic reviews and meta-analysis [1]

Study	AMSTAR-2 Items																Overall Items
	1	2	3	4	5	6	7	8	9	10	11	12	13	14	15	16	
Kang et al., 2019	Yes	No	Yes	Yes	Yes	Yes	No	Partial yes	Yes	No	Yes	Yes	Yes	Yes	Yes	Yes	Critically low
Metha et al., 2023	Yes	Yes	Yes	Yes	Yes	Yes	No	Partial yes	Yes	No	Yes	Yes	Yes	Yes	No	Yes	Critically low
Chan et al., 2023	Yes	Yes	Yes	Yes	No	No	No	Partial yes	Yes	No	Yes	Yes	Yes	Yes	Yes	Yes	Low
Petit et al., 2021	Yes	Yes	Yes	Yes	Yes	Yes	No	Partial yes	Yes	No	Yes	Yes	Yes	Yes	Yes	Yes	Low
Hauer et al., 2019	Yes	No	Yes	Partial yes	Yes	Yes	No	Partial yes	Yes	No	NA	NA	Yes	Yes	NA	No	Critically low
Zhang et al., 2019	Yes	No	Yes	Yes	Yes	Yes	No	Yes	Yes	No	Yes	Yes	Yes	Yes	Yes	Yes	Critically low
Shaheen et al., 2023	Yes	No	Yes	Yes	Yes	Yes	No	Yes	Yes	No	Yes	Yes	Yes	Yes	Yes	Yes	Critically low

Notes: *1*, Did the research questions and inclusion criteria for the review include the components of PICO?; *2*, Did the report of the review contain an explicit statement that the review methods were established prior to the conduct of the review and did the report justify any significant deviations from the protocol?; *3*, Did the review authors explain their selection of the study designs for inclusion in the review?; *4*, Did the review authors use a comprehensive literature search strategy?; *5*, Did the review authors perform study selection in duplicate?; *6*, Did the review authors perform data extraction in duplicate?; *7*, Did the review authors provide a list of excluded studies and justify the exclusions?; *8*, Did the review authors describe the included studies in adequate detail?; *9*, Did the review authors use a satisfactory technique for assessing the risk of bias (RoB) in individual studies that were included in the review?; *10*, Did the review authors report on the sources of funding for the studies included in the review?; *11*, If meta-analysis was performed did the review authors use appropriate methods for statistical combination of results?; *12*, If meta-analysis was performed, did the review authors assess the potential impact of RoB in individual studies on the results of the

meta-analysis or other evidence synthesis?; *13*, Did the review authors account for RoB in individual studies when interpreting/ discussing the results of the review?; *14*, Did the review authors provide a satisfactory explanation for, and discussion of, any heterogeneity observed in the results of the review?; *15*, If they performed quantitative synthesis did the review authors carry out an adequate investigation of publication bias (small study bias) and discuss its likely impact on the results of the review?; *16*, Did the review authors report any potential sources of conflict of interest, including any funding they received for conducting the review?; *NA*, No meta-analysis conducted; *High*, 0–1 noncritical weakness; *Moderate*, > 1 noncritical weakness. The systematic review has more than one weakness, but no critical flaws; *Low*, 1 critical flaw with or without noncritical weaknesses; *Critically low*, > 1 critical flaw with or without noncritical weaknesses.

Table S2. R-AMSTAR scores of the systematic reviews and meta-analysis [2]

Study	R-AMSTAR Items											Overall Items	
	1	2	3	4	5	6	7	8	9	10	11	Score	Quality
Kang et al., 2019	3	4	2	1	2	4	4	3	4	4	3	34	High
Metha et al., 2023	4	4	3	2	2	4	4	4	4	1	3	35	High
Chan et al., 2024	4	1	3	3	3	4	4	4	4	3	3	36	High
Petit et al., 2021	4	4	4	3	2	4	4	3	4	4	4	40	High
Hauer et al., 2019	3	4	4	3	3	4	2	3	1	1	1	29	Medium
Zhang et al., 2019	3	4	4	2	3	4	3	4	4	4	2	37	High
Shaheen et al., 2023	3	2	3	2	2	4	3	4	4	4	3	34	High

Notes: *1*, Was an ‘‘a priori’’ design provided? ; *2*, Was there duplicate study selection and data extraction?; *3*, Was a comprehensive literature search performed?; *4*, Was the status of publication (i.e., grey literature) used as an inclusion criterion?; *5*, Was a list of studies (included and excluded) provided?; *6*, Were the characteristics of the included studies provided?; *7*, Was the scientific quality of the included studies assessed and documented?; *8*, Was the scientific quality of the included studies used appropriately in formulating conclusions?; *9*, Were the methods used to combine the findings of studies appropriate?; *10*, Was the likelihood of publication bias assessed?; *11*, Was the conflict of interest included?; Score interpretation: “Low” (11–22 points), “Medium” (23–33 points), and “High” (34–44 points).

References

1. Shea, B. J., Reeves, B. C., Wells, G., Thuku, M., Hamel, C., Moran, J., ... & Henry, D. A. (2017). AMSTAR 2: a critical appraisal tool for systematic reviews that include randomised or non-randomised studies of healthcare interventions, or both. bmj, 358. https://doi.org/10.1136/bmj.j4008

2. Kung, J., Chiappelli, F., Cajulis, O. O., Avezova, R., Kossan, G., Chew, L., & Maida, C. A. (2010). From systematic reviews to clinical recommendations for evidence-based health care: validation of revised assessment of multiple systematic reviews (R-AMSTAR) for grading of clinical relevance. The open dentistry journal, 4, 84. https://doi.org/10.2174/1874210601004020084
